# Regional Susceptibility to Domoic Acid in Primary Astrocyte Cells Cultured from the Brain Stem and Hippocampus

**Published:** 2008-02-14

**Authors:** Santokh S. Gill, Yangxun Hou, Talat Ghane, Olga M. Pulido

**Affiliations:** 1 Toxicology Research Division, Food Directorate, Health Products and Foods Branch, Banting Research Center, P.L. 2202D2, Tunney’s Pasture, Ottawa, ON, Canada, K1A 0L22 E-mail: Santokh_Gill@hc-sc.gc.ca (S. G.). E-mail: Yangxun_Hou@hc-sc.gc.ca (Y. H.). E-mail: Olga_Pulido@hc-sc.gc.ca (O. P.); 2 Department of Toxicology, Faculty of Pharmacy, Tehran University of Medical Sciences, Enghelab Avenue, Tehran, Iran 14174, P.O. Box: 14155/6451

**Keywords:** Domoic acid, astrocytes, susceptibility, semi-quantitative analysis, electron microscopy

## Abstract

Domoic acid is a marine biotoxin associated with harmful algal blooms and is the causative agent of amnesic shellfish poisoning in marine animals and humans. It is also an excitatory amino acid analog to glutamate and kainic acid which acts through glutamate receptors eliciting a very rapid and potent neurotoxic response. The hippocampus, among other brain regions, has been identified as a specific target site having high sensitivity to DOM toxicity. Histopathology evidence indicates that in addition to neurons, the astrocytes were also injured. Electron microscopy data reported in this study further supports the light microscopy findings. Furthermore, the effect of DOM was confirmed by culturing primary astrocytes from the hippocampus and the brain stem and subsequently exposing them to domoic acid. The RNA was extracted and used for biomarker analysis. The biomarker analysis was done for the early response genes including c-fos, c-jun, c-myc, Hsp-72; specific marker for the astrocytes- GFAP and the glutamate receptors including GluR 2, NMDAR 1, NMDAR 2A and B. Although, the astrocyte-GFAP and c-fos were not affected, c-jun and GluR 2 were down-regulated. The microarray analysis revealed that the chemokines / cytokines, tyrosine kinases (Trk), and apoptotic genes were altered. The chemokines that were up-regulated included - IL1-α, IL-Β, IL-6, the small inducible cytokine, interferon protein 10P-10, CXC chemokine LIX, and IGF binding proteins. The Bax, Bcl-2, Trk A and Trk B were all down-regulated. Interestingly, only the hippocampal astrocytes were affected. Our findings suggest that astrocytes may present a possible target for pharmacological interventions for the prevention and treatment of amnesic shellfish poisoning and for other brain pathologies involving excitotoxicity.

## Introduction

Astrocytes account for 50% of the total cellular volume [1,2] and 90% of the number of brain cells. These cells were traditionally thought to provide structural support for neuronal growth and differentiation and to remove toxic metabolites from the extracellular environment [1,2,3]. The astrocytes are also known to interact with the endothelial cells which form the blood brain barrier (BBB) and regulate the flow of chemicals in and out of the brain [1,2]. In addition to providing structural support, astrocytes also participate in many other processes crucial for the brain through their abundance and diverse ion channels, receptors, and transporters for neurotransmitters and neuromodulation [4-5]. 

Neurotoxicity caused by excitatory amino acids (EAAs) such as glutamate contributes to the pathology of the central nervous system (CNS), including neurodegenerative disease, ischemia and trauma. Glutamic acid and /or its structural analogues such as domoic (DOM) or kainic acid (KA) have been proposed to mediate brain toxicity through their interaction with EAA receptors collectively known as the glutamate receptors (GluRs). Sites often damaged are the hypothalamus by glutamate [6], the hippocampal and neocortical pyramidal neurons by domoic acid [6], the cerebellar Purkinje neurons by ibogaine [6-7] and the corpus striatum by 3-Nitropropionic Acid (3-NPA) and amphetamine [6]. The excitotoxic damage presents as neuronal loss and neuronal degeneration with characteristic cytoplasmic vacuolation and swollen dendrites. The regional distribution of the neuronal lesions reflects that of the GluR distribution. Astrocytes with swollen perikarya and karyorrhexis have often been observed in the vicinity of these lesions [8-9]. It has been suggested that astrocytes contribute to a decline of neurologic function by either the accumulation and release of EAAs after ischemia and oxidative stress or by formation of epileptogenic scars in response to CNS injury and metabolism of protoxins to potent toxins. Recent evidence shows that in response to invasion by microorganisms, both astrocytes and microglia mount an immune response by releasing cytokines [10-13]. Although it is known that astrocytes and microglia are involved in the initiation, maintenance, and suppression of immune responses, they have also been shown to propagate CNS damage [10-13]. Therefore, these cells may also be involved in neurodegenerative disorders. Astrocyte activation (reactive gliosis) and neuroinflammation are known to accompany neurotrauma, stroke, neurodegenerative disease or tumors [14]. Since reactive gliosis represents a homotypic response to diverse insults of the CNS, it is assumed that neuroinflammation is induced by a common set of signals. Cytokines, neurotrophins, and neurohormones are potent biological response modifiers that exhibit a spectrum of cellular actions associated with a variety of neurotoxic exposures that induce neuroinflammation [10,15-17]. The signal transduction pathways and gene-activation events that link these chemical messengers to glial activation are not yet fully characterized. 

Several mechanisms have been implicated as mediators for the effects of DOM [18,19]. These include the potential role played by astrocytes [20]. Acute toxicity studies, using single parenteral doses of DOM, have shown light microscopy evidence of cell injury of the astrocytes in several brain regions [21]. Here we provide additional morphologic evidence of the effects of DOM on astrocytes as assessed by electron microscopy after subchronic oral administration of DOM in rats. We also further explore the pathogenesis of the glial lesion induced by DOM and regional functionality using *in vitro* astrocytes isolated from the hippocampus and the brain stem. Currently, limited information is available on the molecular events altered by DOM that may affect the function of astrocytes. Ultimately, the characterization of the early cellular events subsequent to DOM intoxication will yield new insights for the rational development of novel therapeutic interventions for the prevention and treatment of amnesic shellfish poisoning and for other brain pathologies involving excitotoxicity.

## Materials and Methods

### Animal Experimentation

*Sprague-Dawley* rats (males and females), 34 to 38 days of age, were divided into three groups. These animals were administered daily doses of 0.0, 0.1 or 5.0 mg/kg body weight of domoic acid (Diagnostic Chemicals Ltd) or water (vehicle) by gavage using a flexible feeding tube. Animals were dosed and monitored for 64 days [22]. At the end of the study, the rats were exsanguinated under isofluorane anaesthesia, and perfused with either 10% neutral buffered formalin for light microscopy or 2% glutaraldehyde:2% paraformaldehyde in Tyrode's solution for electron microscopy [23].

### Electron Microscopy

Samples were dehydrated in an increasing alcohol series, cleared with propylene oxide, infiltrated and embedded in epoxy resin. Thin sections from the CA3 region of the hippocampus were mounted on 200 mesh copper grids and stained with uranyl acetate and lead citrate. All observations were made with a Zeiss 902 transmission electron microscope.

### Primary Astrocyte Cell Culture

Astrocytes were cultured from newborn (day one) *Sprague-Dawley *rats as described earlier [23]. Brains were removed aseptically from the pups, minced and mechanically disrupted by vortexing in Dulbecco’s modified Eagle’s medium (D-MEM D) containing penicillin (100 μ/mL) and streptomycin (100 μg/mL). This suspension was passed through a sterile nylon mesh (pore size 70 μm) followed by sieving through 10 μm mesh (Spectrum Labs Inc CA, USA) into D-MEM supplemented with 20% (v/v) fetal bovine serum (FBS-Invitrogen Canada, Burlington. ON, Canada) containing penicillin/streptomycin (100 μg/mL-Invitrogen Canada, Burlington. ON, Canada). Cultures for MTT assays were placed in 96-well plates and seeded at 10^4 ^cells per well. For PCR and gene expression analysis, cells were cultured at 10^5^/ml, in 75 cm^2 ^cell culture flask (Beckon-Dickinson Labware) and incubated at 37 °C in 5% CO_2_ with 95% relative humidity. After 4 days of seeding, the culture medium was replaced with D-MEM containing 10% FBS. Thereafter the medium was changed twice a week. All subsequent experiments were performed on 21-day old cultures. 

### Cell Viability

 Astrocyte cultures (10^4^ per well) in 96 well plates were incubated with 5 different concentrations of domoic acid (0, 0.1, 1.0, 10, 100 and 1000 μM). The effect of DOM on the cell viability was assessed by using MTT uptake assays as described by Mosmann [24]. MTT (final concentration, 0.25 mg/ml) was added to the medium and the cultures were incubated for 3 hrs at 37 °C in 5% CO_2_. The medium was aspirated and the cells were washed twice with PBS and lysed with 100μl dimethyl sulphoxide per well at room temperature. Absorbance was measured at 570 nm, with 630 nm as the reference. The measurements were performed using a microplate reader (Dynatec MR 5000, Dynex Technologies, Chantilly, VA, USA). 

### Neurotoxicology

 For DOM toxicity, the astrocytes were plated at a density of 10^5^ cells/mL in Primaria 75 mL flasks (VWR International Ltd. Missasauga, ON, Canada) and used on day 21. Ten μM of DOM was the optimal concentration for neurotoxicity, as determined in the cell viability assay and this was mixed in the growth medium for 24 hr. For transcriptional analysis, the supernatant was removed, and the cells were trypsinized and washed with PBS and stored at -80 °C until needed. Total RNA was extracted using Trizol Reagent (Invitrogen Canada, Burlington, ON) and stored at -80 °C until analyzed.

###  Microarray****Analysis

The DNA microarray glass slides (RO1) were purchased from Mergen Ltd (San Leandro, CA). The preparation of biotin-labelled cRNA probes was essentially as described by the manufacturer. Briefly, the first and second strand cDNAs were synthesized using a cDNA synthesis kit from Roche Diagnostics Corporation (Indianapolis, IN, USA). The cDNA synthesis was performed according to the manufacturer’s instructions using 10 μg of total RNA and the second strand cDNA was synthesized at 16 °C for 2 hrs. The residual RNA was digested by 15U RNase I at 37 °C for 30 min after ds-cDNA synthesis. Proteinase K 3U was used to eliminate protein contamination. The double stranded cDNA was cleaned by phenol, phenol/chloroform and chloroform sequentially.

To produce the biotin-labelled cRNA probes the ds-cDNA was used as the template. The ds-cDNA was incubated with each of 3 μL rATP (75mM), rGTP (75 mM), rUTP (75 mM), rCTP (15 mM), 10x transcription buffer, T7 enzyme mix, and 4.5 μL biotin-14-CTP (10 mM) (Invitrogen Canada Inc., Burlington. ON, Canada) at 37 °C for 4 hrs. The synthesized cRNA probes were purified using Micro-Spin Columns.

For hybridization, the sheared probe was applied to the sealed hybridization chamber attached to the microarray slide and allowed to hybridize overnight at 30 °C. The slides were removed and washed under both low and high stringency conditions.

The slides were scanned with a Virtek ChipReader (Virtek Vision Corp., Waterloo, ON) and analysed by the Array-Pro program (Media Cybernetics, Silver Spring, MD). The data was transferred to Microsoft Excel for analysis. The genes that had a signal mean ratio which was over 2- fold different in all the three pairs of slides (control and treatment) were further analysed using semi-quantitative PCR. The PCR results were analysed using the AlphaEase program and statistically analysed using the t-test in the Prism 4 software (GraphicPad, Prism 4, San Diego, CA, USA). 

### Semi-Quantitative PCR

 Total RNA was isolated from the primary culture astrocytes using Trizol Reagent (Invitrogen Canada Inc., Burlington. ON, Canada) according to the manufacturer's protocol. The cDNA sequences for the genes of interest were obtained from the National Center for Biotechnology Information (NCBI) and primers were designed using Primer3 software. The synthesis of the primers was carried done by Invitrogen Canada Inc. For RT-PCR analysis, 2 μg of total RNA was used for first strand cDNA synthesis using the Superscript II reverse transcriptase (Invitrogen Canada Inc.) and oligo(dT)_12-18_ primer. The cDNAs were then amplified by PCR using gene specific primers and Taq enzyme (Promega, Madison,WI). The PCR conditions were: denaturation at 94 °C for 1 min, annealing at 55 °C - 60 °C for 1 min, and extension at 68 °C for 1 min. Twenty eight cycles were used for amplification followed by an extension at 68 °C for 10 min. The cDNA products were separated in a 2% agarose gel [25] and were analysed by densiometric analysis using Chemimager software. The internal house keeping gene was 18S (Ambion Inc.). Statistical analysis was done using t-test (GraphPad, Prism 4, San Diego, CA, USA).

## Results

**Figure 1:**
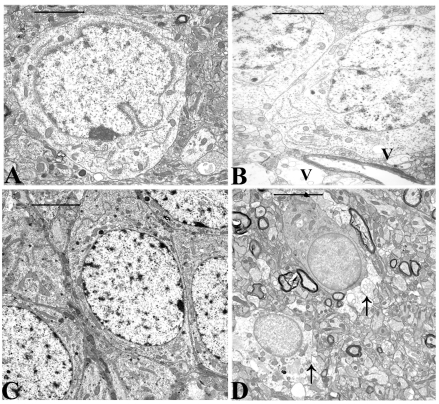
Micrographs from the CA 3 region of the hippocampus of untreated controls and domoic acid treated rats (5.0 mg/kg/day). A). Control animal with well preserved astrocyte-magnification (Bar =1.1μm); B). Astrocyte from treated animal showing minimal vacuolation (V) of the cytoplasm (Barr=1.1 μm); C). Control animal with well preserved pyramidal neurons of the CA 3 region, (Bar=2.5μm)**; **D). Treated animal showing astrocyte cell necrosis (arrow) with disruption of the cytoplasmic membrane and degenerating processes (Bar = 3.5μm).

 Electron microscopy revealed morphological changes in the CA 3 region of the hippocampus of animals treated for 64 days with 5.0 mg/kg/day of DOM as compared to the control and to animals receiving 0.1 mg/kg/bw. Various degrees of degenerating astrocytes from minimal vacuolation of the cytoplasm to necrosis were observed (Figure 1).

**Figure 2:**
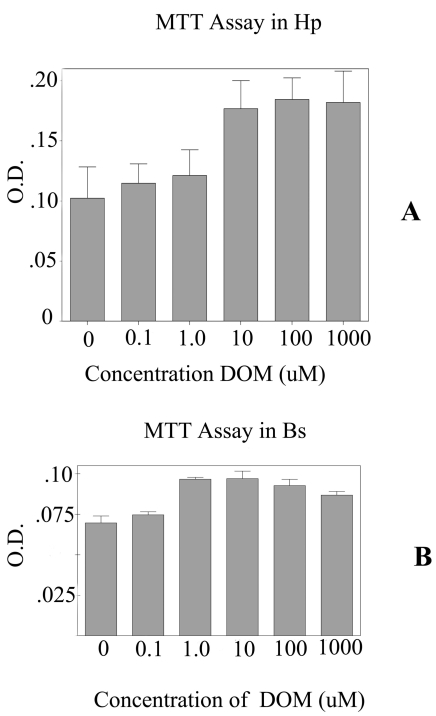
Graph showing the MTT assays for the astrocytes from the A-Hippocampus (Hp) and B- Brain Stem (Bs). Five different concentrations of domoic acid (DOM) were used. 10 μM of DOM was used for neurotoxicology experiments. A). The MTT assays for the astrocytes from the hippocampus (Hp) and B. MTT assays for the astrocytes cultured from the brain stem (Bs). O.D.- optical density.

To further evaluate the effects of DOM on astrocytes, primary astrocytes were cultured from two different regions of the brain focusing on the brain stem and the hippocampus. The MTT assay shows that the astrocytes from the brain stem appeared to be more sensitive to higher (>10 uM) concentrations of DOM than those astrocytes from the hippocampus (Figure 2). Hence, 10 μM of DOM was used for further experiments. After 24 hrs of treatment with DOM, the total RNA was extracted and used for transcriptional analysis. The initial analysis was done on i). the early injury genes (EIGs) including c-fos, c-jun, c-myc, Hsp-72; ii). astrocyte specific markers-GFAP; iii). the glutamate receptor subtypes - GluR 2, NMDAR 1, NMDAR 2A, NMDAR 2B and iv). the proliferating cell nuclear antigen (PCNA).

**Figure 3:**
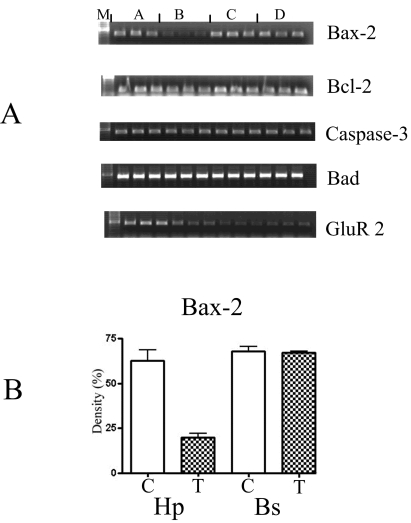
(A) The semi-quantitative analysis of the PCR amplification of different apoptotic markers: Bax-2, Bcl-2, Caspase-3, Bad and GluR 2 derived from RT-PCR performed in triplicate. M: molecular biomarkers; A: control samples from the hippocampus; B: astrocytes treated with 10 μM of DOM from the hippocampus; C: control samples from the brain stem; and D: treated samples from the brain stem. The gels were analysed using the Chemimager and the densities were measured and statistically analysed using the t-test (GraphPad, Prism 4, USA). (B) Shows a graph of the statistical analysis of the marker -Bax-2. C-control, T-astrocytes treated with 10 μM of DOM. Hp- astrocytes isolated from the hippocampus region and Bs- astrocytes isolated from the brain stem. The gels were analysed using the Chemimager and the densities were measured and statistically analysed using the t-test (GraphPad, Prism 4, USA).

The c-jun was down-regulated whereas c-fos and Hsp-72 and the astrocyte specific marker (GFAP) were not affected. The glutamate receptor subtype GluR 2 was down-regulated over 2-fold, (Figure 3), expression of NMDAR 1, 2A and 2B were not detected. These observed changes were only in the astrocytes cultured from the hippocampus. 

**Figure 4:**
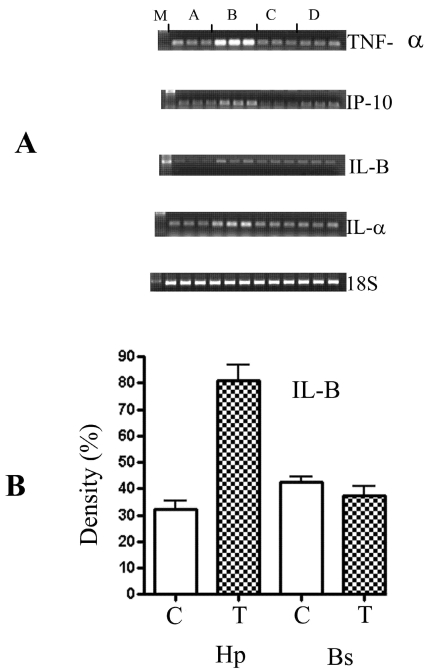
(A) The semi-quantitative analysis of the PCR amplification of different cytokine markers: TNF-α-(tumour necrotic factor), IP-10, IL-B, IL-α, and the house keeping gene 18S (3:7) derived from RT-PCR performed in triplicates. M: molecular biomarker; A: control samples from the hippocampus; B: astrocytes treated with 10 μM of DOM from hippocampus; C: control samples from the brain stem; and D: treated samples from the brain stem. All the reactions were done in triplicate. The gels were analysed using the Chemimager and the densities were measured and statistically analysed using the t-test (GraphPad, Prism 4, USA). (B) Shows a graph of the statistical analysis of the marker -IL-B. C- control, T- astrocytes treated with 10 μM of DOM. Hp- astrocytes isolated from the hippocampus region and Bs- astrocytes isolated from the brain stem. The gels were analysed using the Chemimager and the densities were measured and statistically analysed using the t-test (GraphPad, Prism 4, USA).

To get a global view of the transcriptional changes, three microarrays RO 1 DNA chips were used for each analysis and the putative positive genes were confirmed using semi-quantitative PCR analysis. From the DNA chip analysis, only the chemokines/cytokines, tyrosine kinases and apoptotic genes showed either up- or down-regulated gene expression. This analysis was supplemented with other genes associated with these pathways absent from the DNA chips. The cytokines were all up-regulated significantly as confirmed by the t-tests. These included IL-α, IL-β, IL-6, small inducible cytokine, interferon protein 10P-10, CXC chemokine LIX and IGF binding proteins (Figure 4). Additional genes which were examined including the transforming growth factor (TGF- α and Β), tumor necrosis factor (TNF-α), nerve growth factor (NGF), MIP-1 and IL-7. Of these, only the TNF-α and TGF- β were affected (Table 1). The expression of the other genes were not affected by the exposure to DOM. Other cytokines such as TGF-α , MIP-1, IL-7 and NGF were not induced after exposure to DOM. 

**Table 1:** Biomarkers analysis in the astrocytes after treatment with domoic acid.

Gene (Up-Regulated)	Treatment/Control
TNF-α	2
IL-1α	2
IL-1B	2
IL-6	3
Small inducible cytokine	2
IP-10	2
CXC chemokine LIX	4
CD3	nc
MIP-1	nc
Down-Regulated Biomarkers	
Trk-B	3
Trk-C	3
Bcl-2	2
Bax-2	3
Bad	nc
Caspase-3	nc
TGF-β	3
**Abbreviations:** DOM: domoic acid; TNF: tumor necrotic factor; IL: interleukins; IP 10: interferon-inducible protein 10; TGF: tumour growth factor; Bax; Bcl; Trk: tyrosine protein kinase; MIP: Macrophage inflammatory protein; CD3: cluster of differentiation; nc: no change. The treatment/control represents the densiometric area of the treatment divided by the densiometric area of the controls.	

Two specific membrane receptors, Trk A and Trk B were down-regulated 3-fold, whereas, Trk C was unaffected. In addition, the apoptotic genes- Bcl-2 and Bax were down-regulated 2 and 3 fold, respectively, whereas, Bad and caspase-3 were not affected (Figure 3). 

The oxidation markers- gamma-glutamylcysteine synthetase (GCS), glutathione-S-transferase (GST), glutathione peroxidase (GPx), cyclooxygenase (COX-2), catalase and superoxide dismutase (Zn and Mn dependent) were not affected. 

## Discussion and Conclusions

Although several mechanisms have been implicated on the neurotoxic effect of DOM, the pathogenesis of the glial lesion has received very little attention. This is despite the fact that regardless of the cause of neuronal damage, reactive glial cells always appear at and around the site of degeneration. Earlier studies have shown in addition to the neurons being affected [26], various degrees of injury was observed in hippocampal astrocytes in animals that had received subchronic oral administration of DOM. The present study demonstrated the ultra-structural morphologic changes are similar to those observations reported by light microscopy in acute toxicity studies [21]. In support of the *in vivo* studies, we also conducted *in vitro* studies using primary astrocytes from two different brain regions- the hippocampus and the brain stem. These cells were exposed 24 hrs to DOM and a systematic analysis of the different pathways that have been implicated in neurotoxicity were examined. Two different approaches were used- first, we examined the effects on the known genes that have been reported to be affected by DOM exposure, which included the EIGs, astrocytes specific marker (GFAP), and the glutamate receptors (AMPA and the kainate subtypes). Secondly, we used microarray technology to further elucidate the other pathways that might be involved. All the transcriptional changes were observed only in the astrocytes from the hippocampus and not from the brain stem.

The GFAP biomarker of gliosis [27] was not changed in either of the *in vivo* [22,26] or the *in vitro* studies conducted here. Of the EIG genes, only c-jun was decreased 2-fold, whereas the c-myc, c-fos and the Hsp72 were not altered. Previous studies with neuron cultures have shown that c-fos reaches a peak after 6 hrs of exposure to the stimulus and then declines, whereas c-jun is activated after 24 hrs of exposure and is followed by the induction of Hsp-72 [28,29]. The lack of altered expression in GFAP and some of the EIGs, is simply due to the fact that the expression of these is transient and measurements were done outside of the relevant time window [28,29].

 DOM is structurally similar to the excitatory neurotransmitter glutamate and glutamate is known to exert its affect by binding to and activating the ionotropic glutamate receptors (iGluRs) including the subtypes NMDAR 1, GluR1, GluR 2, GluR 5, and GluR 6 in the neurons. Metabotropic receptors are not involved in DOM toxicity [30]. In our study with astrocytes, only GluR 2 was increased 2-fold, whereas no expression was detected for any of the NMDAR subtypes.

 From the microarray analysis and confirmed by semi-quantitative analysis, the chemokines / cytokines, tyrosine kinases (Trk), and apoptotic genes showed either up- or down-regulated gene expression. Several pro- and anti-inflammatory cytokines including TNF-α, IL-1α, IL-1B, IL-6, CXC chemokine LIX, interferon protein 10P-10, small inducible cytokine and IGF binding protein were up-regulated 2x fold or higher. However, not all cytokines were altered since cytokines are regulated in cascades. The IL-1Β and TNF-α are known to initiate the early events which are pleiotrophic and trigger general responses that are not specific for the initiating stimulus or antigen [15] and these were both up-regulated. The cytokines act by binding to specific membrane receptors, which signal the cell via second messengers, such as the tyrosine kinases (Trk), to alter its gene expression. The Trk A and B were down-regulated 3-fold, whereas, Trk C was unaffected. The apoptotic genes, Bcl-2 and Bax were altered, whereas, BAD and caspase-3 were not affected. The R01 chip also had other neural markers including the different ion channels such as potassium and sodium, γ-aminobutyric acid, cholinergic and the serotonin receptors. None of these were changes.

The oxidation stress markers are known to occur at the site of inflammation, these can also contribute to damage. The activation of astrocytes can lead to the expression of inducible nitric acid oxide synthase (inos), superoxides and nitric oxide which are involved in the removal of free radicals. In our study, the GSTα, GCS, catalase, superoxide dismutase (Zn and Mn dependent), inducible nitric oxide (inos) and COX-2 were not altered after 24 hrs exposure to DOM. 

In our study the injury to the astrocytes was characterized by ultra-structural changes, inflammatory,immuno, growth factor, cytokines, and changes in the cell death genes. It has been shown that the activation of the astrocytes is an important defence mechanism. Localized production of cytokines and chemokines has been shown in many debilitating neurologic disorders such as multiple sclerosis, Alzheimer's disease and AIDS [12,16,31]. As the brain ages, inflammatory events become less well-regulated and hence environmental exposure can exacerbate the endogenous heightened CNS inflammation [32]. Many chemokines are capable of directly regulating signal-transduction pathways that are involved in a variety of cellular functions, which range from synaptic transmission to growth. Clearly, the potential use of chemokines and their receptors as targets for therapeutic intervention in CNS disease might now have to be considered in the context of the broader physiological functions of these molecules. Therefore, an understanding of the different interactions between inflammatory mediators leading to restricted local reaction is important in the development of novel therapeutics for inflammatory CNS diseases [33,34,35]. This is facilitated further by the fact that astrocytes from different regions show functional heterogeneity. Recently, Tomita* et al*. [36] showed that the spice curcumin strongly inhibited the transcription of the cytokine MIP-2 which is mainly produced by the astrocytes. Ananth *et al.* [37] demonstrated that DOM-induced astrogliosis and neurodenegeration were reduced after 5 days of melatonin administration. Hence, it might be possible to use anti-inflammatory intervention to slow down the progression of many different forms of neurodegenerative diseases.
